# Living Lab Dementia: process evaluation of an academic-practice partnership in German long term care for people living with dementia - study protocol

**DOI:** 10.1186/s12877-024-05649-z

**Published:** 2025-01-18

**Authors:** Andrea Leinen, Felix Bühler, Sascha Köpke, Mareike Löbberding, Gabriele Meyer, Helga Schneider-Schelte, Stefan Wilm, Anja Bieber, Martin N. Dichter

**Affiliations:** 1 Institute of Nursing Science, Faculty of Medicine and University Hospital Cologne, University of Cologne, Gleueler Straße 176-178, 50935 Cologne, Germany; 2 Institute of Health and Nursing Sciences, University Medicine Halle, Medical Faculty, Martin Luther University Halle-Wittenberg, Magdeburger Straße 8, 06112 Halle (Saale), Germany; 3 German Alzheimer Association, Keithstraße 41, 10787 Berlin, Germany; 4 Institute of General Practice, Heinrich Heine University, 40225 Moorenstrasse 5, Düsseldorf, Germany

**Keywords:** Process evaluation, Complex intervention, Logic model, Academic-practice partnership, Living lab, Dementia, Mixed methods, Long-term care

## Abstract

**Background:**

Living Labs, as a type of academic-practice partnerships, possess the potential to transform care and research into a participatory partnership and narrow the research-practice gap to improve evidence-based and Person-centred care. Given the lack of systematic investigations of Living Labs in healthcare, we will establish a dementia-specific academic-practice partnership (*Living Lab Dementia)* in Germany and conduct a process evaluation. The aim of this study is to gain insights into the intervention itself (mechanisms of impact) and its implementation process (degree of implementation, barriers, and facilitators).

**Methods:**

This process evaluation of the multi-center research project PraWiDem (German acronym for linking professional nursing practice and research in dementia) will be conducted applying a convergent mixed methods design and will be based on the Medical Research Council (MRC) framework for the development and evaluation of complex interventions. The intervention is presented in a logic model which describes relevant basic theoretical assumptions, intervention components, implementation aspects, mechanisms of impact, relevant outcomes, and the context in which the intervention is delivered. Data will be collected before the intervention (T_0_), during the intervention period, and at follow-up after 18 months (T_1_). Qualitative data will be collected through semi-structured interviews and focus groups. Quantitative data sources will be process documents and questionnaires.

**Discussion:**

Our study will provide important insights into the *Living Lab Dementia* intervention and its implementation processes. The results of this evaluation will contribute to the refinement of the intervention and its implementation processes, and will enable to measure the impact of these processes in future studies.

## Background

Evidence-based practice is commonly known as the key to provide best possible care [1]. Even though this has been known for several years, evidence-based practice is still not comprehensively implemented in care settings [2]. Despite a growing body of knowledge, it currently takes many years for the latest research findings to be translated into care practice [3]. This phenomenon is commonly known as the “research-practice gap” [4]. It is caused by various factors, including the clear separation between researchers and practitioners, which is visible in their areas of work as well as in their opportunities for collaboration [5]. In our experience the separation between academic professionals and practitioners in Germany is notably pronounced in the field of long-term care (LTC), although the demand for LTC is increasing due to the ageing population and the surge in chronic and degenerative diseases [6, 7]. Today, there are approximately 1.8 million people living with dementia in Germany [8]. In LTC settings, ambulatory and residential care, challenges in the care of people living with dementia exist and can place substantial stress for both care staff and people living with dementia [7, 9]. National and international strategies dealing with the best possible care (i.e. evidence based practice and Person-centred care) for people living with dementia therefore call for greater collaboration between research and practice as well as the involvement of people living with dementia and their relatives in research processes [7, 10].

Academic-practice partnerships are promising innovative concepts that have the potential to promote care and research in participatory partnerships and to foster knowledge circulation for the improvement of evidence-based practice [11]. So called Living Labs, one form of an academic-practice partnership, employ user-centered approaches to integrate research and transformational processes in real-life settings [12, 13]. Living Labs in healthcare are not physical spaces, but interdisciplinary partnerships involving researchers, patients or care dependent people, their representatives, caregivers, facility managers, and educators [14]. User centeredness and co-creation are key elements [13], whereby the latter has been shown to bridge the gap between research and practice more effectively through knowledge circulation than often used unidirectional knowledge transfer strategies, characterized by a passive flow of information (from producer to user) [15, 16]. Integrating co-creation and participation makes it more likely to produce acceptable, valuable, and sustainable results for practice [15, 17].

First introduced in the field of urban planning and design, Living Labs have been adopted in various areas and are now increasingly implemented into healthcare, especially in the care for older people [18]. The “Living Lab in Ageing and Long term care” at Maastricht University in Netherlands serves as a successful example of such a collaboration in LTC and has operated for 25 years with currently over 180 participating facilities [19]. Their “Limburg Living Lab” model has two key elements: interdisciplinary collaboration working on research projects (including care receivers and their representatives) and so-called Linking Pins (healthcare professionals and researchers working together in the care organization) [14]. LTC in Germany is characterized by a lack of academic-practice partnerships. Therefore, in the three-year federally funded project PraWiDem, we will adapt the “Limburg Living Lab” model [14, 19] to the German LTC context with a specific focus on dementia care [20].

As Living Labs involve different stakeholders, settings and components and are implemented in a dynamic context, they should be considered a complex intervention [21]. To our knowledge, this was not taken into account in previous projects [14, 22–27]. Therefore, we will conduct a process evaluation, guided by the MRC framework for the development and evaluation of complex interventions [21].

## Objectives

During the implementation of our *Living Lab Dementia* intervention we aim to describe:

- the degree of implementation (dose, reach, fidelity and adaptations).
- mechanisms of impact within the intervention, and
- barriers and facilitators for the implementation as well as the implementation context (micro-, meso-, and macro- levels).

## Methods

### Design

**Fig. 1:**
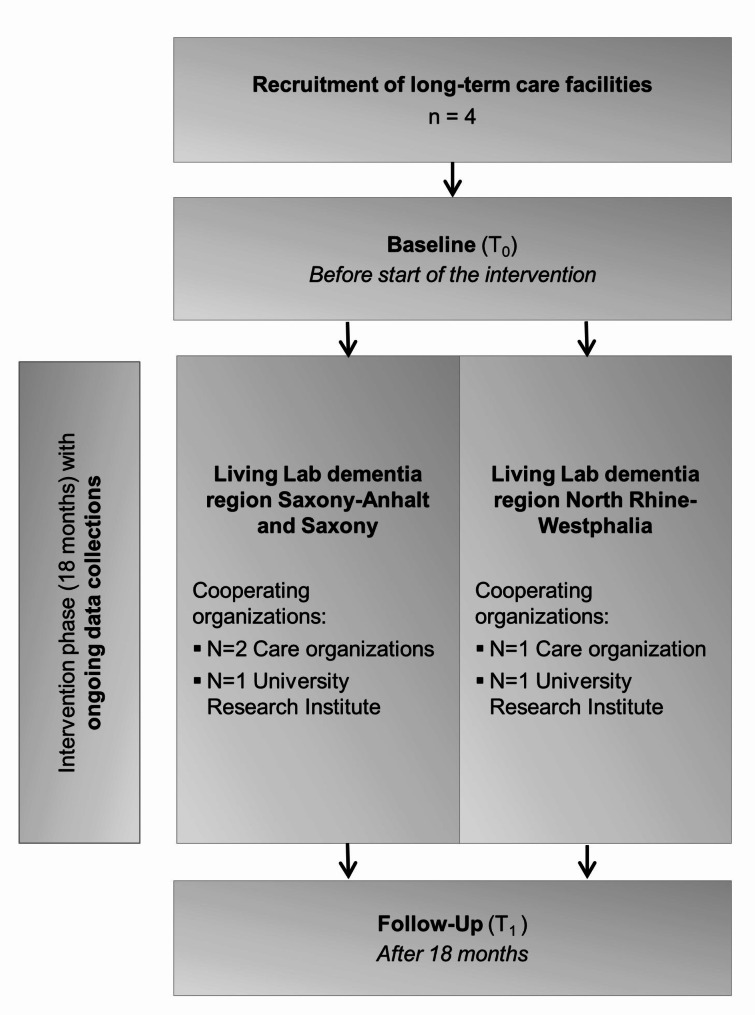
Timeline overview

The process evaluation is based on a mixed methods design with several measurement points: Before the intervention (T_0_), ongoing during the intervention time, and at follow-up after 18 months (T_1_) (Fig. 1). A convergent mixed methods design will be used to obtain valid results and gain a more complete understanding of the complex intervention. Therefore, qualitative and quantitative data will be collected and triangulated [28].

### Setting

**Fig. 2:**
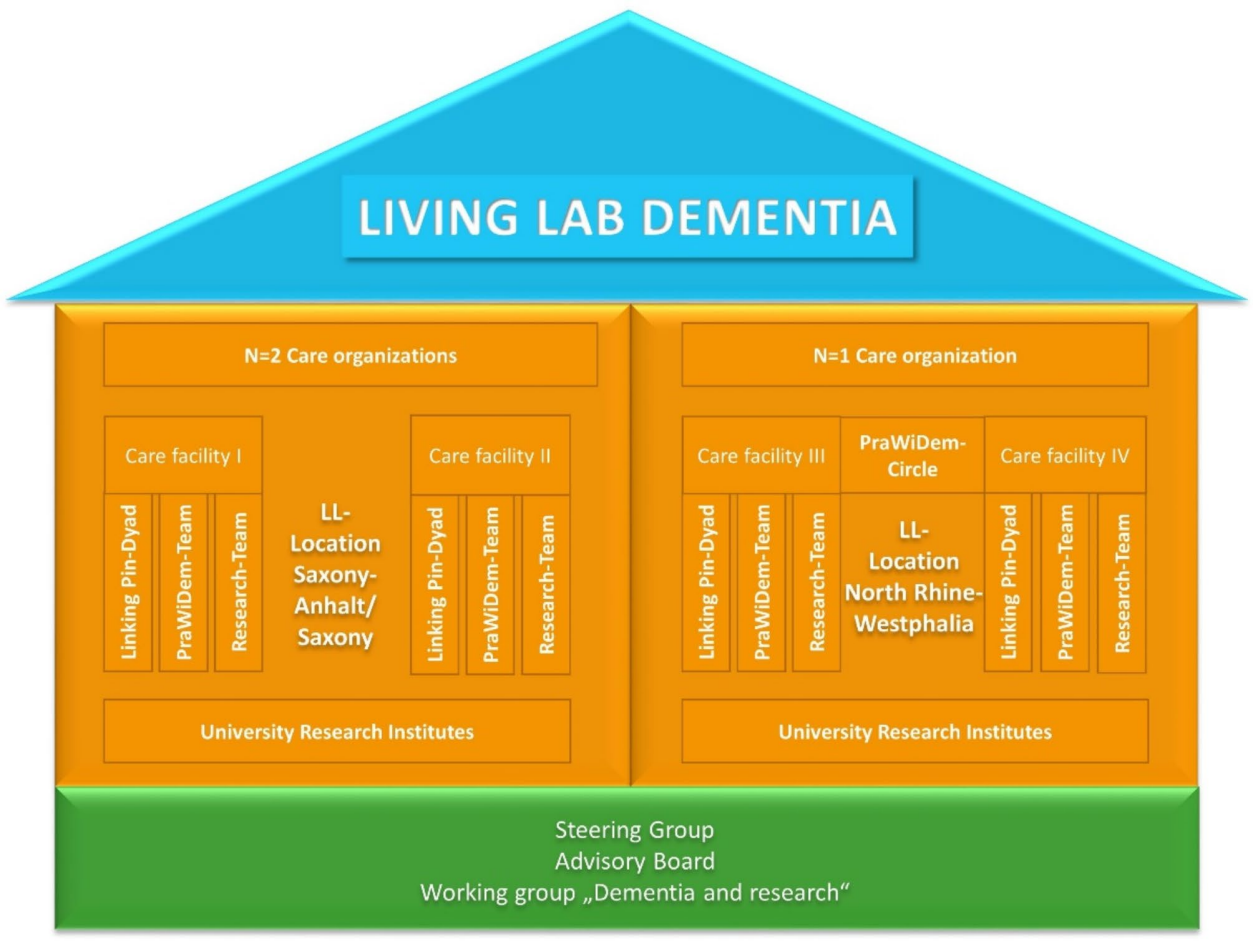
Structure of the *Living Lab Dementia* LL = Living Lab

This study will be conducted at two different locations in the east and the west of Germany. At each location, the intervention will be carried out by university-based research institutes (see description of research team) and two practice partners from ambulatory and residential LTC (Fig. 2). At the Living Lab location in Saxony/Saxony-Anhalt, one of the two care facilities is a small-scale facility for people living with dementia. Currently 20 employees care for a group of 30 people living with severe dementia. The other facility is an ambulatory care service where 15 employees are caring for about 80 clients. At the Living Lab location North Rhine-Westphalia, the two facilities apply a community-based caring system, providing care for both ambulatory and residential care clients by the same staff. With this community-based approach, people living in the community can participate in activities or take part in meals at the care facility. Combined, both facilities employ around 245 staff members who provide care for approximately 175 residential care clients and 175 ambulatory clients.

### Intervention

**Fig. 3:**
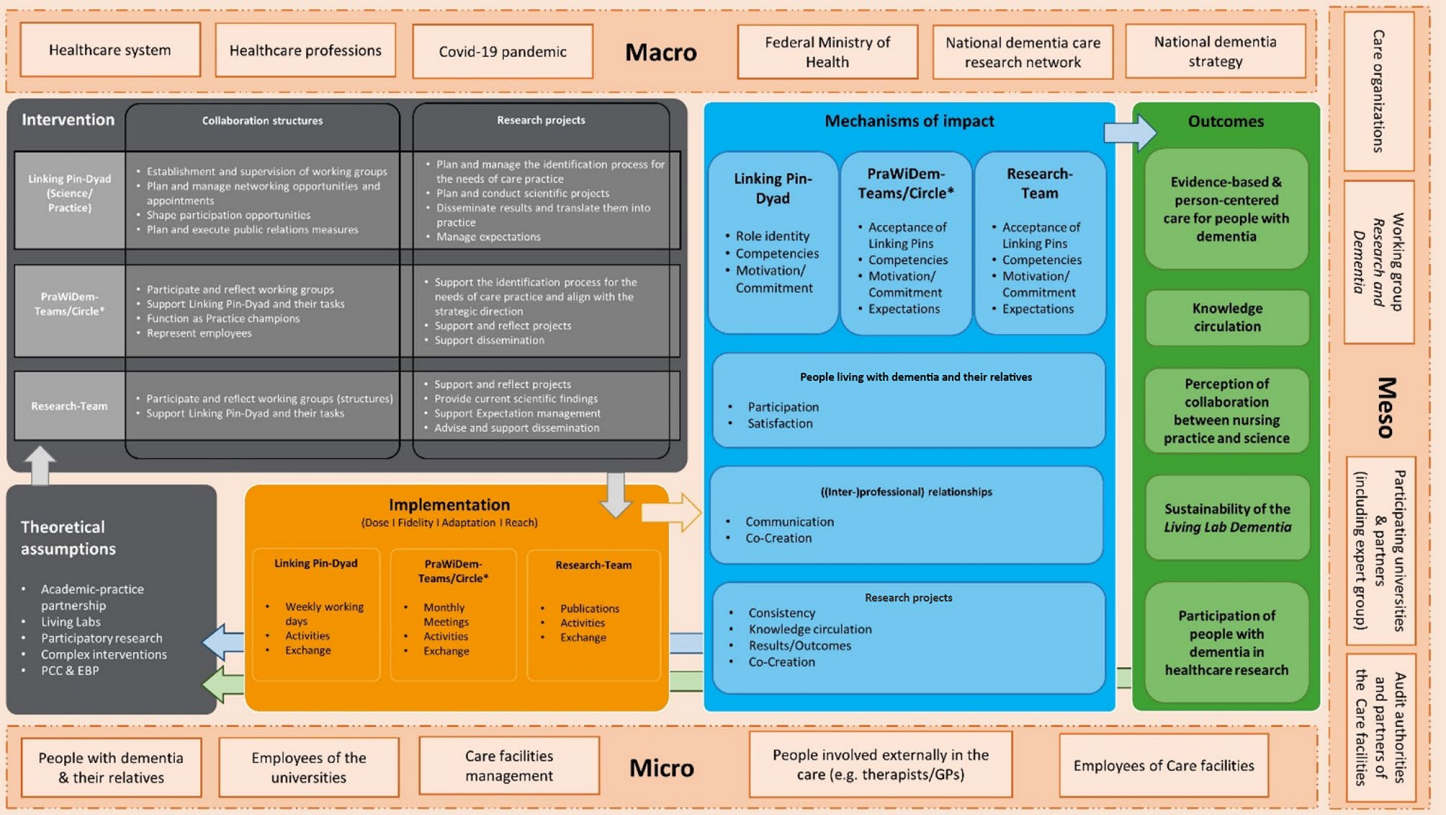
Logic model of the *Living Lab Dementia*. *if several facilities of the same organization are involved, there are exchange structures at the organizational level (PraWiDem circle) and the facility level (PraWiDem team); EBP = Evidence-based Practice; GP = General practitioner, PCC = Person-centred Care

The adaptation of the intervention was based on the preliminary results of a scoping review [29], an interview study [30], and several meetings with researchers from Maastricht University, the project advisory board, and different stakeholders from the partner organizations. To visualize and clarify the intervention delivered, a program theory was developed, as recommended by the MRC framework [21]. This is presented as a logic model guided by the W.K. Kellogg Foundation [31], considering the recommendations by Moore [32]. The logic model describes relevant basic theoretical assumptions, intervention components, implementation aspects, mechanisms of impact, relevant outcomes, and the context in which the intervention is delivered [31, 32] (Fig. 3).

The *Living Lab Dementia* intervention comprises three components: (1) the Linking Pin Dyad, (2) the PraWiDem teams of the care facility respectively the PraWiDem circle of the participating care organizations, and (3) the research team. Each component works on two different levels of action: the collaboration structures and the research projects. Research projects arise from topics within the care organization, whereby it is not specified whether each facility must have its own topic or not. However, it is important that the topic will be developed in close collaboration with the care providers. The intervention components for *Living Lab Dementia* has been adapted as described below:

#### Linking pin dyad

Linking Pins, as a specific structural element of the Living Lab, seem particularly important, their role being comparable to so-called change agents, which are regarded as crucial for the successful change process in practice [33]. Practice Linking Pins primarily need a strong interest in the collaboration [30] between care practice and research and the ability to devote the expected time commitment of one day per week for the project. A completed three-year nursing training or a comparable qualification is required, as well as practical working experience in the LTC of people living with dementia. In-depth knowledge of structures and processes within the participating facility is required. Further qualifications (e.g. further training in geriatric psychiatric care), academic qualification (e.g. bachelor’s degree), and experience in project work or in implementing change processes are desirable. Willingness to participate in project-related further training is also beneficial. Scientific Linking Pins also need a distinct interest in the collaboration between nursing practice and research. At least master’s degree in health, nursing, or social sciences (or a related field) is essential, accompanied by proficient knowledge of clinical-epidemiological research methods and evidence-based practice. Additionally, candidates must have completed a three-year nursing training or possess an equivalent qualification, along with practical work experience. The role of the Scientific Linking Pins requires the ability to work independently in a scientific field and to collaborate and communicate with different target groups.

#### Team/circle members

If several facilities of the same organization are involved, there are exchange structures at the organizational level (PraWiDem circle) and the facility level (PraWiDem team). The members of the PraWiDem team should be interested in the project and motivated to participate. They may possess any qualification and belong to any professional group involved in the care of people living with dementia in the participating LTC facilities. The members of the PraWiDem circle will be Linking Pin-Dyads, representatives of the PraWiDem team and people who are involved in the partnership across all institutions, e.g. project leaders from the University, Chief Executive Officers, representatives of facility management, project managers of the organization, work councils and representatives of other facilities within the organization.

#### Research team

The research team will consist of members from the participating universities. Through the participation of the Martin Luther University Halle-Wittenberg and the University of Cologne, qualifications for nursing professions are represented at all levels (from research associates, senior researchers and full professors). The Heinrich Heine University Düsseldorf additionally provides a person (full professor) to represent general practitioners’ perspectives.

The intervention components are described in more detail in Table 1 following the TIDieR checklist [34, 35].

**Table 1:** Detailed description of the *Living Lab Dementia* intervention

No.	What? Procedures	Why? Rationale and theory	What? Material	How? Modes of delivery	Who? Intervention provider	When and how much?
1.	**Implementation of a Linking Pin Dyad per care facility** *Collaboration structures*: ● Establishment and supervision of working groups ● Plan and manage networking opportunities and appointments (e.g. team-, circle meetings, annual Living Lab Meetings) ● Shape participation opportunities ● Plan and execute public relations measures *Research projects*: ● Plan and manage the identification process for the needs of care practice ● Plan and conduct the research projects ● Disseminate results and translate them into practice ● Manage expectations	● Crucial for the co-creative work between research and practice ● Change agents are recommended for the implementation of complex intervention [33, 38, 39] ● Insights into the care facilities [40] ● Building meaningful connections [40]	● Appropriate workplace ● Software packages (e.g. Microsoft office) ● Release from regular activities in the work schedule once a week ● Information material: poster, flyer, online presence, articles, presentations, pocket cards etc. ● Promoting material: pencils, bags, lanyards, sweets, etc.	● Working day together in the care facility ● Meetings with different stakeholders ● Appointments with overall care staff	Scientific Linking Pin: ● Research associate (M.A.; M.Sc.) or research fellow (post-doc) ● Vocational training in a healthcare profession or practical work experience Practice Linking Pin: ● Vocational training in nursing care or a therapeutic field ● Employment in the direct care of people living with dementia ● In-depth knowledge of structures and processes within the long-term care facilities	● Once a week preferably regular daily working time (approx. 8 h)
2.	Implementation of a Team/Circle* per care facility/organization *Collaboration structures*: ● Participate and reflect working groups ● Support LP-Dyad and tasks ● Function as practice champions ● Represent employees *Research projects*: ● Support the identification process for the needs of care practice and align with the strategic direction ● Support and reflect projects ● Support dissemination	● Integration of different roles and qualifications ● Practice champions are recommended for the implementation of complex intervention [39, 40]	● Conference room ● Information material: poster, flyer, online presence, articles, presentations, pocket cards etc. ● Work schedule that favors joint working hours	● Meetings with different stakeholders	● 3–6 motivated employees of different professions and qualifications ● Care facility manager (all levels) ● Possibly further interested stakeholder from quality management, works council, other care facilities, general practitioners, therapists	● Once a month/quarterly meetings (between 1 and 2 h)
3.	**Establishing a fixed collaboration with a research team per location** *Collaboration structures*: ● Participate and reflect working groups and processes ● Support LP-Dyad and tasks *Research projects*: ● Support and reflect projects ● Provide current scientific findings ● Support expectation management ● Advise and support dissemination	● For effective knowledge circulation there is a need for an established long-term collaboration	● Access to libraries and scientific papers ● Information material: scientific poster, articles, presentations	● Research time ● Meetings with different stakeholders	● Employees of cooperating universities from different levels of qualification (full professors, senior researcher, research associates)	● Once a week

*if several facilities of the same organization are involved, there are exchange structures at the organizational level (PraWiDem circle) and the facility level (PraWiDem team); **LP** = Linking Pin

### Sampling, participants and recruitment

As recommended for a convergent design, sampling for the quantitative and qualitative data will be identical [28, 36]. In this study, sampling will be strongly influenced by the collaborative structure as one part of the intervention. Recruitment of the participants for key roles in intervention components (described in Intervention chapter) will determine the sample [37]. Overall, participants for the process evaluation will mainly be recruited through participating organizations.

Linking Pins will be recruited through both, public and internal job advertisements. Recruitment of team members will be carried by Linking Pins through various strategies, primarily by distributing flyers on bulletin boards and conducting project presentations at each facility. In addition to the participants determined by the key roles in the intervention components, further study participants will be people living with dementia and, if required, their relatives. During the first phase of the PraWiDem project, a group of people living with dementia (Working group *Dementia and Research)* was established in collaboration with the German Alzheimer Association to ensure that people living with dementia have the opportunity to participate. The group includes 5 people living with dementia, who will participate and advise the research projects. They were recruited through the collaboration with the German Alzheimer Association and its existing network.

### Data collection and management

The logic model will guide data collection. Hence the degree of implementation, mechanisms of impact within the intervention, and barriers and facilitators for the implementation as well as the implementation context (micro-, meso-, and macro- levels) will be assessed. The degree of implementation will be measured considering four different dimensions: reach, dose, fidelity and adaptation [32] (Table 2). All relevant factors will be recorded on cluster level (Care facilities) and overall. Qualitative data will be collected through semi-structured interviews (face-to-face or online) and focus groups (face-to-face or online). Quantitative data sources will be process documents and questionnaires (socio-demographic data and facility characteristics). In order to obtain valid and solid data, objectivity, reliability and validity will be considered for quantitative data. For qualitative data, credibility, authenticity, integrity, congruence and sensitivity will be applied [41]. Strategies for quality assurance include that for both strands, the data collection instruments will be jointly developed and critically reflected in the project team. Pre-testing the data collection templates of the quantitative data and testing the interview guidelines for the qualitative data further will also contribute to the quality of the data [28].

**Table 2:** Overview of data collection strategies

Domain	Dimension	Aims	Target group	Data collection methods	Data type	Measurement point
Implementation	Dose	Present number of intervention components and participants overall and within the clusters on both levels (Collaboration structure & Research projects)	n.a	Document Analysis	Quantitative	ongoing
		Present the number of exchanges (within the components, overarching (including People living with Dementia and relatives & external)	n.a	Document analysis		
		Present the number of public relations measures	n.a	Document analysis		
	Reach	Describe profiles of participants filling the targeted positions within the components	n.a	Document analysis	Quantitative	T0 + ongoing
		Present reasons for participation/non-participation	SLPs/PLPs Teams/circles Research team	Questionnaire (1)		
		Present recruitment processes	n.a	Document analysis		
	Fidelity	Describe to which extent the intervention components, at both levels (Collaboration structure & Research projects), are implemented	SLPs/PLPs Teams/circles CEOs Research team	Individual interview Focus groups Document analysis	Quantitative + Qualitative	ongoing + T1
		Describe the support requirements of the Linking Pin-Dyad	SLPs/PLPs Teams/circles CEOs Research team	Individual interview Focus groups	Qualitative	T1
		Describe project planning process	SLPs/PLPs Research team	Individual interview		
	Adaptation	Describe adaptation processes and reasons for adaptation	SLPs/PLPs Research team	Individual interview	Qualitative	T1
Mechanisms of impact	Intervention components	Identification of mechanisms of impact in the areas role identity, competencies, motivation/commitment at the level of Linking Pin-Dyad	SLPs/PLPs Teams/Circles Research team	Individual interview Focus groups	Qualitative	T1
		Identification of mechanisms of impact in the areas acceptance of Linking Pin role, competencies, motivation/commitment and expectations at the level of teams/circle/ Research team	SLPs/PLPs Teams/Circles Research team	Individual interview Focus groups		
	People living with dementia and their relatives	Describe the ways to participate for people living with dementia (and their caregivers, if applicable)	SLPs/PLPs Working group *Dementia and Research* Involved People living with Dementia (and poss. their relatives)	Individual interview Focus groups	Qualitative	T1
		Describe the satisfaction with the participation opportunities	Working group *Dementia and Research* Involved People living with Dementia (and poss. their relatives)	Individual interview Focus groups		
	(Inter-) professional relationships	Identification of mechanisms of impact in the areas communication and participation	SLPs/PLPs Teams/Circles Research team	Individual interview Focus groups	Qualitative	T1
	Research projects	Identification of mechanisms of impact in the areas of consistency, knowledge circulation, results/outcomes and co-creation	SLPs/PLPs Teams/Circles Research team	Individual interview Focus groups Document analysis	Quantitative + Qualitative	ongoing + T1
Contextual factors	Micro-, Meso-, Macro-Level	Describe factors of micro-, meso-, macro-level that facilitate or hinder the implementation of intervention components	SLPs/PLPs Teams/Circles CEOs Research team	Individual interview Focus groups	Qualitative	T1
		Describe characteristics of the organizations and care concepts of the practice partners (ambulatory and residential care)	n.a.	Questionnaire (2)	Quantitative	T0

**CEO** = Chief Executive Officer, **SLP** = Scientific Linking Pin, **PLP** = Practice Linking Pin

Data will be collected and stored pseudonymously and will be anonymized as quickly as possible (e.g. interview data after the transcription). Identification lists and paper-based documents will be stored separately from other study material and kept locked away. All collected data will only be accessible and deleted by members of the project team. The audio recordings of interviews will be transmitted to a cooperating transcription office via password-protected files for the purpose of transcription. Access to these files will be restricted to one research assistant at the respective study center. Data protection follows the current data protection laws in Germany.

### Data analysis

According to the convergent design, quantitative and qualitative data will be analyzed separately after data collection [28]. In a following step, we will triangulate these results as described below (result-based integration) [42].

Quantitative data (process documents and questionnaire data) will be analyzed descriptively using IBM SPSS Statistics [43]. Therefore, a standard operating procedures will be developed. One researcher will analyze the data, with at least 10% of the data being independently verified by a second researcher. Qualitative data (interview and focus group data) will be recorded and transcribed verbatim and analyzed by conducting a structuring content analysis using a deductive-inductive approach described by Kuckartz [44] based on the logic model. The analysis process comprises seven phases: initiating text work, development of the main categories (often using the interview guide), coding of the material using the main categories, collection of the text passages according to categories, inductive determination of subcategories, coding using the differentiated category system and, finally, simple and complex analysis with visualization [44]. The data will be reviewed by one researcher, who will present the in between results of each step regularly to the project team and discuss them critically. These steps can also be carried out by two researchers at the same time. For coding and analysis the software MAXQDA [45] will be used.

After the quantitative and qualitative data have been analyzed, common concepts will be identified in the results and arrayed together so that they can be easily compared. These will also be presented graphically, e.g. by common display tables. Disconfirming results will be identified, transparently presented and discussed in the project team. Finally, the congruencies and inconsistencies will be interpreted to gain a deeper insight into the research questions [28].

### Dissemination

The main results of the process evaluation will be published in an international, peer reviewed journal and will be presented at relevant scientific conferences. Results will be reported based on this study protocol as well as the recommendations of the CReDECI 2 [46] and COREQ [47] reporting guideline. Authorship will be shared by those involved in the study according to the current guidelines of the International Committee of Medical Journal Editors (ICMJE) [48]. People who are primarily involved in care, as well as people living with dementia and their relatives, do not always have access to international journals and may struggle with language barriers. Therefore, additional target group-specific methods and materials (e.g. flyers and presentations) will be used to ensure that all participants have access to the results of this study. Results of the research projects conducted within *Living Lab Dementia* will be presented in the partner care organization and published separately. Further information could be retrieved on the project homepage (https://www.umh.de/prawidem).

## Discussion

PraWiDem applies an adaptation of the “Limburg Living Lab” Model [19] for the first time in the German healthcare system, additionally tailoring the approach to a dementia specific target group. Therefore, our goal is to concretize and examine the intervention and its implementation process in a way that it can serve as a blueprint after the project period.

Although measurable outcomes are considered in the logic model, this study will focus on the investigation of the *Living Lab Dementia* intervention itself and its implementation processes. The results of this evaluation will contribute to the refinement of the intervention and its implementation processes, and will enable to measure the impact of these processes with regard to an outcome evaluation in future studies. This compatible with the phases of the MRC Framework [21].

A deviation from other academic-practice partnerships, including the “Limburg Living Lab” model [14], is the current positioning of scientific Linking Pins. Due to many years of experience and the number of participating facilities, in the “Limburg Living Lab” model [19] Scientific Linking Pins work together with several facilities of an organization. In the *Living Lab Dementia*, each Scientific Linking Pin will collaborate with no more than two facilities. This approach aligns with the recommendations gathered during the adaptation of the Living Lab to implement the intervention on a small scale. It will provide Scientific Linking Pins in the early stage of the partnership with the opportunity to build a peer group and exchange ideas and experiences. Additionally, it will ensure a division of tasks within the project team in the interests of practicality, as most of them will have two roles within the project. Moreover, this will correspond to the geographical circumstances of the participating organizations, so the Scientific Linking Pin can assure one working day per week in the facility.

The above-mentioned overlap of the researchers’ roles will be a challenge of the study. The researchers responsible for the process evaluation will partly also have the role of scientific linking pins. During the process evaluation, it will be important to limit role overlap as far as possible, especially during data collection. Therefore, the Scientific Linking Pins will only be involved in data collection if necessary and will not interview the Practice Linking Pins of their partnering facility. Close communication with the other researchers is important, who represent a neutral point of view throughout the data collection and analysis process.

The results will be made available to other interested organizations (e.g. at universities with a focus on nursing science or LTC organizations) for the initiation of further Living Lab locations throughout Germany.

## Data Availability

No datasets were generated or analysed during the current study.

## Abbreviations

LTC: Long-term care
MRC: UK Medical Research Council
